# A Molecular and Co-Evolutionary Context for Grazer Induced Toxin Production in *Alexandrium tamarense*

**DOI:** 10.1371/journal.pone.0015039

**Published:** 2010-11-29

**Authors:** Sylke Wohlrab, Morten H. Iversen, Uwe John

**Affiliations:** 1 Department of Ecological Chemistry, Alfred Wegener Institute for Polar and Marine Research, Bremerhaven, Germany; 2 Department of Geosciences/Marum, University of Bremen, Bremen, Germany; Lund University, Sweden

## Abstract

Marine dinoflagellates of the genus *Alexandrium* are the proximal source of neurotoxins associated with Paralytic Shellfish Poisoning. The production of these toxins, the toxin biosynthesis and, thus, the cellular toxicity can be influenced by abiotic and biotic factors. There is, however, a lack of substantial evidence concerning the toxins' ecological function such as grazing defense. Waterborne cues from copepods have been previously found to induce a species-specific increase in toxin content in *Alexandrium minutum*. However, it remains speculative in which context these species-specific responses evolved and if it occurs in other *Alexandrium* species as well. In this study we exposed *Alexandrium tamarense* to three copepod species (*Calanus helgolandicus*, *Acartia clausii*, and *Oithona similis*) and their corresponding cues. We show that the species-specific response towards copepod-cues is not restricted to one *Alexandrium* species and that co-evolutionary processes might be involved in these responses, thus giving additional evidence for the defensive role of phycotoxins. Through a functional genomic approach we gained insights into the underlying molecular processes which could trigger the different outcomes of these species-specific responses and consequently lead to increased toxin content in *Alexandrium tamarense*. We propose that the regulation of serine/threonine kinase signaling pathways has a major influence in directing the external stimuli i.e. copepod-cues, into different intracellular cascades and networks in *A. tamarense*. Our results show that *A. tamarense* can sense potential predating copepods and respond to the received information by increasing its toxin production. Furthermore, we demonstrate how a functional genomic approach can be used to investigate species interactions within the plankton community.

## Introduction

Dinoflagellates of the genus *Alexandrium* possess a high ecological impact due to their ability to form Harmful Algal Blooms associated with Paralytic Shellfish Poisoning (PSP). PSP is a threat to marine aquaculture and shellfish consumers, occurring worldwide with increasing frequency and distribution [1]. PSP is caused by an accumulation of highly potent neurotoxic alkaloids, the Paralytic Shellfish Toxins (PST), in the marine food web. Accumulations of PST can induce mass death of fish [2], [3], high mortalities among marine mammals [4], [5], and cause human intoxication via consumption of contaminated shellfish [6].

Saxitoxin is one of ∼two dozen naturally occurring PST and was first discovered in 1975 [7]. Despite the early discovery of the first PST, the ecological function that PST may play in marine dinoflagellates remains unclear [3]. It has been suggested that they serve a function in nitrogen storage [8], possess pheromone activity [9], have an impact on associated bacteria [10], and act as defense compounds [11]. Yet, PST have been ruled out in allelopathic interactions, including interactions with heterotrophic micrograzers in plankton food webs [12], [13]. One may therefore not exclude the fact that a single metabolite may have multiple ecological functions. If we consider metabolic energy costs associated with biosynthesis and modification of secondary metabolites, it seems intuitive that natural selection would favor metabolites with multiple functions [14]. Several investigations have suggested that the PST function as defense compounds against copepods [reviewed in 15]. However, studies focusing on the influence of copepod grazing on dinoflagellates have shown both (1) high ingestion rates of toxic *Alexandrium* with no adverse effects on the grazers as well as (2) enhanced mortality of the grazer [16], [17]. Thus, it seems that grazing experiments are highly dependent on the *Alexandrium* strain investigated, as well as on the grazer species [11]. Parallel investigations concerning the effects of toxin producing cyanobacteria on zooplankton grazers are described for freshwater ecosystems [18]. *Microcystis* spp. strains, for example, can differ significantly in their toxin content [19] and the potential predator (*Daphnia* spp.) shows different levels of impairment upon grazing, potentially due to differences in detoxification abilities or adaptations to the toxins following post-exposure [20], [21]. Also, naturally occurring *Alexandrium* populations are composed of different strains, producing different amounts of PST, and possessing different PST profiles [22]. Such genotypic and phenotypic diversity could lead to varying results among grazing studies. Furthermore, it has been shown that some copepods can detoxify PST [23]. It has also been observed that copepods, which have been historically exposed to *Alexandrium* blooms, are less affected by PST compared to copepods originating from regions devoid of *Alexandrium* [24]. Further, some copepods are able to adapt to the toxins within a few days of exposure and can develop resistance towards the PST [25], while other studies observed selective feeding on non-toxic *Alexandrium* over toxic cells by copepods [26]. However, the selective feeding only seems to apply when there is high cell concentration of *Alexandrium* [27]. The range of observed adaptations by copepods exposed to PST suggests a predator-prey co-evolution, and may support the hypothesis that PST acts as defense compounds. Additional evidence of PST acting as grazer defense compounds is given by Selander et al. [28], who demonstrated that PST content was increased in *Alexandrium minutum* after exposure to waterborne cues from copepods, which correlated with a decreased copepod grazing. Further, Bergkvist et al. [29] found that *A. minutum* only increased its PST production significantly when exposed to waterborne cues from two out of three different copepods, which indicates that toxin production might be target specific.

This study aimed to investigate the effects on PST content in *Alexandrium tamarense* when exposed to copepod grazing or the waterborne cues from copepods. By collecting the copepods from the same geographic regions as the origin of the *A. tamarense* strain used, we assumed that the copepods had a history of co-existence with *A. tamarense*. It has been suggested that co-existence is an important driver for the chemical cue specific responses [29]. Assuming that one of the ecological functions of PST is defense against predation, exposure of *A. tamarense* cells to actively grazing copepods or only their waterborne cues should induce an increase in PST production by *A. tamarense.* The impact of PST on the copepods, post exposure to *A. tamarense,* was estimated by relating their internal PST concentration, ingestion rates and behavioral response to the toxins. The response of *A. tamarense* after exposure to copepods and potential waterborne cues was assessed via screening of gene expression patterns through microarray analyses for all treatments and related to the PST measurements. This functional genomic approach allowed us to trace cue perception to changes in gene expression, since potential waterborne cues recognized by *A. tamarense* may alter gene expression through receptor stimulation.

## Results

### Cellular PST content of *A. tamarense*

**Figure 1:**
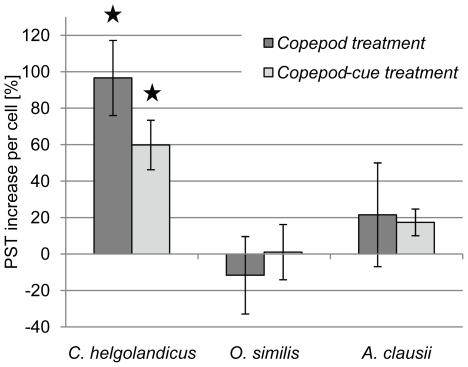
PST content of *A. tamarense* cells after 48h exposure to live copepod and/or waterborne cues. Differences in PST content are denoted in percentage compared to that of the control. Bars are grouped according to the respective copepod species tested. The first bar of each group represents the treatment with copepods, the second bar represents the treatment with the copepod waterborne cues and the third bar represents the control treatment (n = 3 each). Bars marked with asterisks show a significant higher PST content compared to the control (ANOVA, *p<0.05*).

Exposure to *C. helgolandicus* individuals and water borne cues caused the largest increase in *A. tamarense* PST content per cell compared to the control (Figure 1 and Table 1). Incubation with *C. helgolandicus* individuals raised the cellular PST level of *A. tamarense* by 100%, and incubation with waterborne cues from *C. helgolandicus* resulted in 60% higher PST content per cell, compared to that of the control incubations. The differences between the three treatments were significant (ANOVA: n = 3; *p*<0.05) whereby both the test treatments differed significantly from the control treatment, and also from each other (Holm-Sidak method: *p*<0.05). In contrast, no significant change in total PST content of *A. tamarense* was observed when exposed to *O. similis* individuals or *O. similis* waterborne cues (ANOVA: n = 3; *p*>0.05). Similarly after exposure to *A. clausii*, no significant changes in cellular PST concentrations of *A. tamarense* could be verified (ANOVA; n = 3; p>0.05).

**Table 1:** Cellular PST content of *A. tamarense* in pg cell-1 after 48 h exposure to different copepod species.

PST[pg cell-1]	*C. helgolandicus*	*O. similis*	*A. clausii*
***Copepod treatment***	59.74±6.26*	14.60±3.51	23.53±5.51
***Copepod cue treatment***	48.57±4.12*	16.70±2.50	22.72±1.42
***Control***	30.39±3.24	16.52±1.24	19.36±1.41

Asterisks indicate significant changes in PST content compared to the control (ANOVA, *p<0.05*).

### Ingestion rates and PST content of copepods

**Figure 2:**
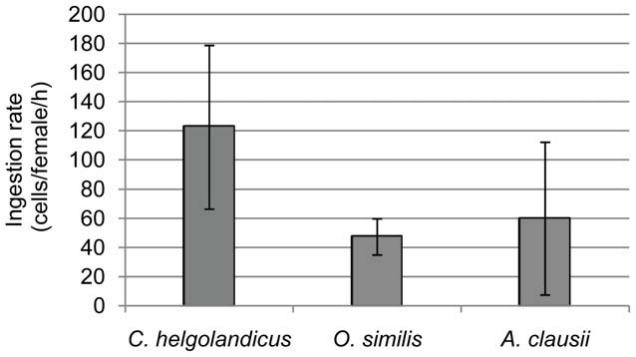
Ingestion rates of the copepod species determined after 48 h. Ingestion rates calculated after Frost (1972) and standard deviations, reported as ingested cells per copepod species per hour (*C. helgolandicus n = 30, O. similis n = 90 and A. clausii n = 45*).

**Figure 3:**
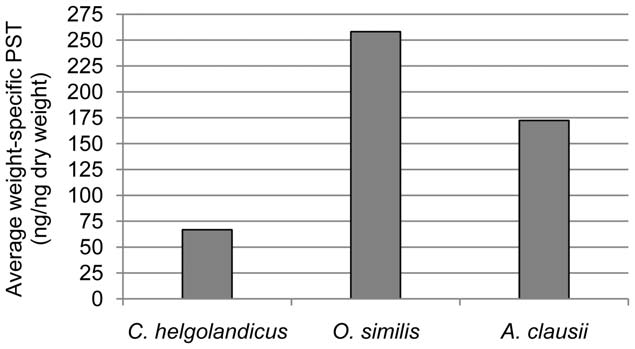
Average PST content of the copepod species determined after 48 h. PST measurements taken out with pooled copepods (excluding dead individuals) of every group (*C. helgolandicus n = 24, O. similis n = 67 and A. clausii n = 25*). Dry weight of copepods was determined through interpolation using the equations of Uye [67].

*C. helgolandicus* had two folds higher average ingestion rates of *A. tamarense* cells compared to the average ingestion rates of both *O. similis* and *A. clausii*, which had ingestion rates of ∼50 and ∼60 cells per female per hour, respectively (Figure 2). Though no significant differences due to high standard deviations could be verified for the determined ingestion rates (ANOVA: *p*>0.05). The lowest weight-specific PST content was found in *C. helgolandicus,* which had an average weight-specific PST content 3.7 and 2.5 times lower than the average weight-specific PST content for *O. similis* and *A. clausii*, respectively (Figure 3).

### Behavioral response of copepods

**Figure 4:**
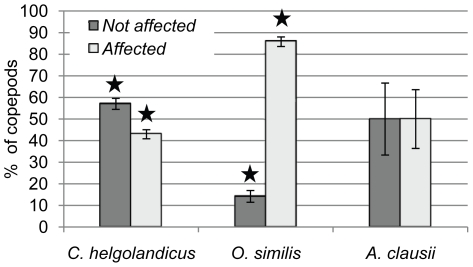
Copepod condition after 48 h exposure to *A. tamarense* cells. Results are grouped according to species (*C. helgolandicus n = 30, O. similis n = 90, A. clausii n = 45*). The first bar displays the percentage of individuals with no noticeable change in their response behavior (not affected). The second bar represents the percentage of disoriented and dead individuals (affected). Asterisks mark significant differences in the amount of non affected and affected individuals (Students *t-*test, n = 3 *p*<0.05).

A significantly higher proportion of unaffected copepods was only observed within the *C. helgolandicus* individuals (Students *t-*test, n = 3 *p*<0.05). After 48h incubation with *Alexandrium* cells, 57% of the *C. helgolandicus* individuals showed an escape response, interpreted as normal response behavior, while 29% showed no escape response and 14% of *C. helgolandicus* died during the incubation. In contrast, *O. similis* individuals revealed a significantly higher proportion of affected copepods (Students *t-*test, n = 3 *p*<0.05): 86%. Here, the group of affected copepods is composed of 65% individuals with no escape response and of 21% dead individuals. No noticeable change in escape behavior and therefore no effect were observed for 14% of the *Oithona* individuals. *Acartia* individuals exposed to *A. tamarense* showed an equal mean distribution of not-affected and affected copepods (50% each). Due to the standard deviation no significance could be determined (Students *t-*test, n = 3 *p*>0.05) (Figure 4). Affected individuals were composed of 10% with no escape response and 40% dead copepods.

### Gene expression profiles in *Alexandrium*

**Figure 5:**
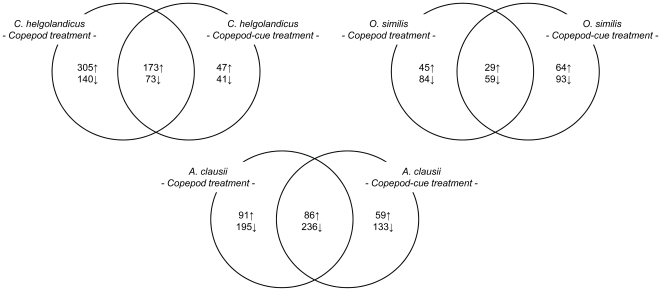
Venn diagram of responsive ESTs after 48 h exposure to different copepod species and cues. Numbers of responsive ESTs are separated in up (↑) and down (↓) regulated ESTs. The intersections displays the number of ESTs regulated in both treatments. Identification of regulated ESTs is based on microarray hybridizations and evaluated with a Student's *t-*test against a control treatment with n = 3 and p<0,05.

A significant change in the gene expression compared to the control treatment was observed for every treatment (Student's *t*-test against control treatment; *p*<0.05; Figure 5). Additionally, overlaps in responsive ESTs could be identified for every treatment with copepod individuals and respective waterborne cues. The highest numbers of up-regulated genes were observed in the treatments containing *C. helgolandicus* individuals and *C. helgolandicus* waterborne cues whereas the lowest numbers were observed in the treatments containing *O. similis* individuals and *O. similis* waterborne cues. Numbers of up-regulated genes observed from the *A. clausii* treatments were intermediate. Regarding the numbers of down-regulated genes, numbers observed for the treatments with *C. helgolandicus* and *A. clausii* individuals and waterborne cues exceeded numbers achieved for the *C. helgolandicus* waterborne cue treatment and the *O. similis* treatments.

**Figure 6:**
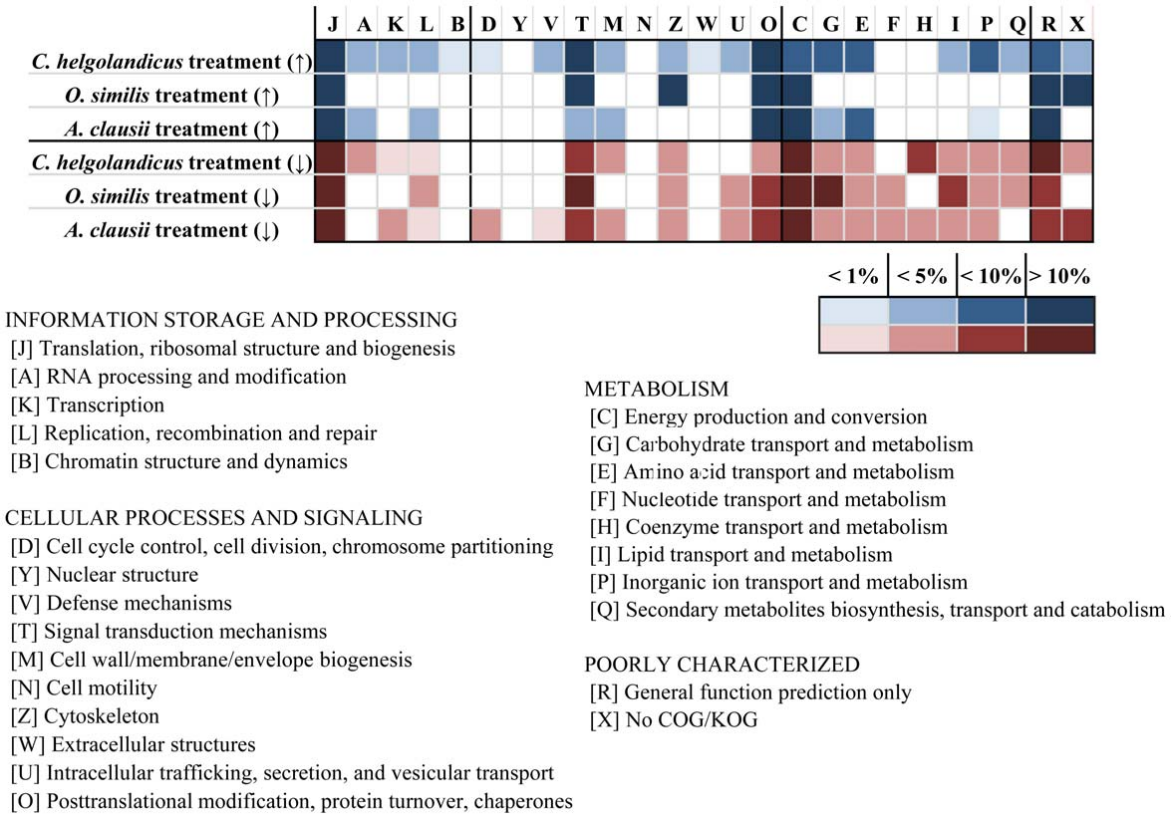
KOG category distributions of differential expressed ESTs identified through microarray hybridizations. The table displays the KOG category distribution of up (↑) and down (↓) regulated ESTs in comparison to a control treatment. The color strengths displays the amount of ESTs per group calculated in percent from total ESTs grouped into KOGs with known or general function prediction.

The distribution of differential regulated genes from the different copepod treatments based on annotations according to KOG categories indicate a broad response concerning multiple cellular function (Figure 6). A full list of the regulated genes with annotations can be retrieved from the supplemental material for the *Calanus*, the *Oithona*, and the *Acartia* treatment, respectively (Table S1, S2, S3).

The difference in expression of main signaling proteins, the serine/threonine kinases, as well as further proteins containing calcium-binding domains is highlighted in Table 2. Up-regulation of serine/threonine kinases was observed in *Alexandrium* exposed to live *C. helgolandicus*, leading to an increased expression of 8 serine/threonine kinases, 3 of them possessing and calcium dependent activity. Down-regulation of serine/threonine kinases was also observed for the *C. helgolandicus* and for the *A. clausii* treatment (Table 2). In contrast, the treatment containing *O. similis* individuals resulted in no difference regarding the expression of serine/threonine kinases. Altered expression of transcripts encoding for proteins containing calcium-binding domains were triggered by exposure to all live copepod species investigated in this study.

**Table 2:** Differential regulated genes coding for proteins with serine/threonine kinase activity and/or calcium binding domains.

Treatment	Regulation	Probe identifier	Putative gene product	Serine/threonine kinase	Ca^2+^-dependent activity	Fold change
***C. helgolandicus***	***upregulated***	*CUST_322_PI235167212*	*Mitogen-activated protein kinase kinase*	**x**		1.9
		*Amin_forward_07528*	*ERK activator kinase*	**x**		3.7
		*Amin_forward_00243*	*Mitogen-activated protein kinase ERK-A*	**x**		1.7
		*Amin_forward_03821*	*Serine/threonine protein kinase*	**x**		1.7
		*CUST_6797_PI235167212*	*Serine/threonine protein kinase*	**x**		1.5
		*CUST_2821_PI235167212*	*Calcium-dependent protein kinase*	**x**	**x**	1.5
		*CUST_4965_PI234877732*	*Calcium-dependent protein kinase*	**x**	**x**	3.2
		*Amin_forward_04046*	*Calcium-dependent protein kinase*	**x**	**x**	1.6
		*Amin_forward_04727*	*Calmodulin*		**x**	1.7
		*CUST_1488_PI234877732*	*C2 domain containing protein*		**x**	2.0
		*CUST_16_PI234877732*	*C2 domain containing protein*		**x**	1.6
		*Amin_forward_01439*	*Efhand containing protein*		**x**	1.6
		*CUST_9039_PI235167212*	*Efhand containing protein*		**x**	2.2
		*CUST_6674_PI234877732*	*Calcium-dependent protein kinase*	**x**	**x**	1.6
	***downregulated***	*CUST_9538_PI234877732*	*Efhand containing protein*		**x**	1.6
		*CUST_4186_PI234877732*	*C2 domain containing protein*		**x**	3.2
		*Amin_forward_09119*	*Calcium-dependent ion transporter*		**x**	1.7
		*CUST_5223_PI234877732*	*cAMP-dependent protein kinase rs*	**x**		1.7
***O. similis***	***upregulated***	*CUST_5675_PI234877732*	*Efhand containing protein*		**x**	1.5
	***downregulated***	*CUST_5852_PI234877732*	*C2 domain containing protein*		**x**	1.9
		*CUST_5506_PI234877732*	*Efhand containing protein*		**x**	1.6
		*CUST_8137_PI235167212*	*Calmodulin*		**x**	1.7
		*Amin_forward_09189*	*Efhand containing protein*		**x**	1.7
***A. clausii***	***upregulated***	*Amin_forward_10237*	*Protein kinase*	**x**		1.5
	***downregulated***	*Amin_forward_04046*	*Calcium-dependent protein kinase*	**x**	**x**	1.6
		*Amin_forward_04729*	*Calmodulin*		**x**	2.1
		*CUST_6892_PI234877732*	*Calmodulin*		**x**	2.0
		*CUST_9187_PI234877732*	*Calreticulin*		**x**	1.6
		*CUST_691_PI235167212*	*Efhand containing protein*		**x**	2.0
		*CUST_7538_PI234877732*	*Efhand containing protein*		**x**	2.4
		*Amin_reverse_15532*	*Efhand containing protein*		**x**	1.6
		*CUST_6400_PI234877732*	*Efhand containing protein*		**x**	2.1
		*CUST_6496_PI234877732*	*Efhand containing protein*		**x**	1.7

## Discussion

The present study showed clear differences in the response of *Alexandrium* to different copepod species. These differences were evident on both the level of PST content of the *Alexandrium* cells as well as on the molecular level as indicated by differential gene expression patterns. Furthermore, the toxins produced by *A. tamarense* showed different effects on the tested copepod species, resulting in different internal PST content and different levels of affections of the toxins between the copepod species.

*Alexandrium tamarense* showed the highest increase of PST content when exposed to *C. helgolandicus* waterborne cues and/or individuals. Despite this finding, *C. helgolandicus* had the highest ingestion rates among the tested copepods, and the lowest weight-specific internal PST levels. In addition, *C. helgolandicus* was the least affected species of the tested copepods in this study, therefore we interpret that these results indicate a predator-prey relationship in which both, predator and prey show evidence of co-evolutionary processes. The high ingestion rate, low influence on fitness, and low weight-specific internal PST levels of *C. helgolandicus* indicate an adaptation through detoxification mechanisms that lead to decreased sensitivity to PST. Detoxification mechanisms in copepods have been observed in previous studies [30], [31]. The increased cellular PST level in *A. tamarense* for both, the treatment with live *C. helgolandicus* and only their cues, suggests a magnified defense response towards a predator capable of handling PST and thus posing a potentially higher risk for the prey population. The significant higher PST content in *A. tamarense* exposed to live *C. helgolandicus* compared to only their cues may be due to continuous release of potential alarm signals from wounded *A. tamarense* cells and chemical compounds from the copepods. Opposite, the amount of cues that could trigger PST production in the treatment with only waterborne cues will either remain constant or decrease through e.g. degradatory processes during the 48 h incubation. Taken together, the magnified defense response of *A. tamarense* against *C. helgolandicus* and the latter's potential ability to detoxify PST indicates that *Calanus* is a historical threat to natural *A. tamarense* populations. High grazing impacts of *Calanus* on *Alexandrium* have previously been found during an *in situ* study [32], and support the potential predator-prey co-evolution between the two species.

*Alexandrium* cellular PST level did not respond to *O. similis* waterborne cues and individuals, indicating the *A. tamarense* did not view *O. similis* as a potential grazer. This was also found by Turner [32] who observed low grazing impact from *O. similis* on *A. tamarense*. Despite no changes in PST production, *O. similis* was still the most affected copepod in our study and had the highest weight-specific internal PST levels. Thus, *O. similis* does not seem to have any resistance or detoxification mechanisms for PST, which is supported by Saiz et al. [33] who found that *Oithona* is very sensitive to toxins. The grazing by *O. similis* observed in this study might have occurred due to *A. tamarense* offered as the sole diet, since *Oithona* present *in situ* avoids the ingestion of toxic *Alexandrium* [32]. It is possible that *Oithona* might have evolved a prey avoidance strategy when alternative diets are available, rendering it sensitive to even moderately low PST production. Consequently, there is no need to enhance the PST production in *Alexandrium*. Exposure to *A. clausii* waterborne cues and individuals only slightly raised the internal PST content of *A. tamarense*. Mean ingestion rates, internal PST content as well as the proportion of affected *A. clausii* individuals were intermediate compared to the *C. helgolandicus* and *O. similis* treatments.

Avery and Dam [25] described individual PST resistance in the copepod *Acartia hudsonica*. Their results suggest that naturally occurring populations of *A. hudsonica* possess different resistant phenotypes frequencies. They further suggested that there is a substantial cost involved with resistance for the phenotype involved, and therefore no allele fixation occurs within a single population. If similar mechanisms are present in *A. clausii* copepods, this might explain the high standard deviations regarding the fitness of the individuals. Additionally, different frequencies of resistant copepods in the replicate treatments could account for high variations in the internal PST measurements of *A. tamarense*. Higher numbers of unaffected, grazing copepods could indicate a higher predation risk of *A. tamarense* and, thus, raise internal PST content.

The results of this study support the findings of Selander et al. [28], concerning increased toxin content in *A. tamarense* after exposure to waterborne cues from copepods. Similar to the observations for *A. minutum* by Bergkvist et al. [29], we observed that increased cellular toxin levels in *A. tamarense* upon exposure to copepods and their cues were target specific. It has been suggested that PST production evolved as a defense towards copepod species which pose a threat to *A. minutum* under natural conditions [29]. Our findings support this hypothesis, since (1) not all of the tested copepod species triggered an increased toxin production, and (2) toxin production seems to correlate with both the presence of and cues from copepods that had high grazing impact on *A. tamarense*. The internal toxin content of *A. tamarense* as a defense mechanism seems to be dependent on the potential grazing impact that a given copepod species has on the population of *A. tamarense.*

The continuous production of PST in *A. tamarense* may lead to a permanent basic protection against copepods which did not evolve detoxification abilities and consequently may lead to grazing avoidance strategies. This might also apply for *Oithona* and *Acartia* which are ambush feeders [34], [35] and are therefore able to feed more selectively than *Calanus*, which is a filter feeder [36].

Necessary for this adaptive response to different copepod species is that *A. tamarense* recognizes a specific copepod as a threat. Although mechanisms controlling adaptive responses in phytoplankton have not been thoroughly studied, Laforsch et al. [37] proposed that unspecific alarm cues from conspecifics may in fact indicate a predation risk in the cladoceran *Daphnia*, and activate inducible defense. White dotted mosquito pupae have been shown to associate novel predatory odors, with alarm cues released from injured conspecifics, thus developing a response solely to the predator cues [38]. The ability of phytoplanktonic organisms to gather specific information through chemical signals concerning abiotic and biotic properties of their current surroundings and to respond appropriately to this information is indeed remarkable [reviewed in i.e. 39]–[41]. Even resting cysts are able to recognize the presence of grazers [42]. With respect to these sensing capacities, such mechanisms like ‘threat sensitive learning’ could have evolved the grazer-specific response toward copepod species that present a threat (e.g. *C. helgolandicus*). Hence, the species-specific alarm cues are not activated when non-threatening copepods are present, e.g. *O. similis.*

On a molecular level this means that *Alexandrium* must have receptors transmitting the received signals into the cell where they are processed, and finally induce the expedient response to the received infochemicals. Such receptors comprise fast evolving proteins that are coupled to signal cascades and are capable of adapting to new or changing environmental cues in a relatively short period of time through slight mutations of an ancestor receptor [43]. Despite the lack of knowledge regarding these processes of adaptive receptor radiation in phytoplankton, their existence is evidenced by, i. e. the presence of adaptive feeding receptors (C-type lectins) [44], enabling food particle selection in the marine dinoflagellates *Oxyrrhis marina* [45]. Thus, the existence of adaptive and therefore tunable receptors could have evolved in *Alexandrium* to integrate waterborne cues from the environment and to respond appropriately.

Chemical signals generated by live copepods and their waterborne cues induced a change in *Alexandrium* gene expression with observed overlapping responsive genes in the respective copepod/copepod-cue treatment for every species tested. Thus, the observed gene expression patterns indicate that the presence of all three tested copepod species and their cues was sensed by *A. tamarense*, yet elicited a different response (see Figure 4). Hence, when the ability of *A. tamarense* to recognize the presence of a copepod is solely based on PST level changes in *Alexandrium* it cannot be identified if the lack of increased PST production is due to no recognition of the copepod or if the copepod is recognized but not viewed as a potential risk.

For the interpretation of the results gathered from the microarray analyses we assume that the sum of regulated genes reflects those genes, for that altering the expression rate is required to integrate the external stimuli into the current stable cellular state, i.e. cell cycle progression [46]. Thus, grazing from copepods triggers gene regulation for a larger range of genes and not only the genes directly involved in defense. This process is indicated by the distribution of regulated genes in different functional categories observed in this study (Figure 5). Such complex responses are also known from plants being attacked by herbivores. After being attacked, herbivory-specific signals are generated by the plant and further converted to large-scale biochemical and physiological changes through complex networks [47], [48]. A very prominent and adaptive signaling event is the phosphorylation of different target proteins through serine/threonine kinases [49]. The changing number of these kinases over time is often the result of a feedback regulation from the integration of signals stimulating receptors [49]. Altered transcriptions levels of protein kinases are known to trigger the responses of plants towards herbivore attack [48], [50]. Vardi et al. [51] showed that changes in the activity, abundance, and expression of protein kinases are involved in the competitive interactions between the cyanobacterium strain-specific perception of *Microcystis* and the dinoflagellate *Peridinium gatunense*.

Different expression rates of serine/threonine kinases observed in this study suggest that these serine/threonine kinases are involved in the different responses of *A. tamarense* to the received copepod-cues (see Table 2). Three of the serine/threonine kinases that were differentially expressed in the *C. helgolandicus* treatment are grouped (based on annotations) into the class of mitogen-activated protein kinases (MAPKs) and respective downstream kinases (MAPKKs) and four are grouped into the class of calcium dependent protein kinases (CDPKs). In the *A. clausii* treatment, one kinase could be grouped into the class of CDPKs whereas no MAPK or CDPK was among the regulated genes in the *O. similis* treatment. MAPK cascades are known to be an important pathway downstream of receptors regulating cellular responses to several external stimuli and are evidenced to be involved in regulating defensive responses through jasmonic acid formation after wounding in tobacco species [52]. An activation of CDPKs and MAPKs after attack by a generalist herbivore has been described for *Nicotiana attenuate* and *Solanum nigrum* [53]. Additionally, an accumulation of transcripts coding for MAPKs and CDPKs as part of the defensive response in *N. attenuate* is described in Wu et al. [50]. In the present study, the treatment with *C. helgolandicus* inducing the highest increase in internal PST levels of *A. tamarense* showed the strongest change in altering expression levels of MAPKs and CDPKs transcripts. A direct involvement of MAPKs and CDPKs in coordinating the response towards copepod grazers in *A. tamarense* should therefore be considered.

Besides CDPKs, further transcripts of proteins with a dependency of calcium ions as second messengers have been identified as differently expressed in this study. The regulation of intracellular calcium concentrations and oscillations is a well-described mechanism to transduce environmental changes into the cell [reviewed in 54,55]. Also in marine phytoplankton, several studies concerning Ca^2+^ signaling highlight their importance in responding to environmental changes [54], [55]. The transcriptional regulation of genes coding for proteins possessing a calcium dependent activity might be another important trigger in tuning the different responses at the cellular level towards copepod grazers in *A. tamarense*.

### Conclusions

This study demonstrates that increased PST production in *A. tamarense* is inducible through the presence of copepod grazer in a species-specific manner and that these specific responses are detectable through functional genomic approaches. The role of PST in acting as defense compound should therefore be reconsidered with respect to genotypic variation and phenotypic plasticity of *Alexandrium* species and tested copepod grazers. Since the present study investigated the effect of one clonal strain of *A. tamarense* on the copepod species we cannot exclude different outcomes of the same study when using different strains. The treatments with copepods or copepod-cues resulting in altered gene expression patterns can be considered as an outcome of the ongoing dialog between *Alexandrium* and its environment and the result of integrating and processing the received information into the most appropriate response selected through evolutionary processes. Different abundances of MAPKs together with CDPKs and further Ca^2+^-dependent proteins potentially influence the overall cell response through directing the external stimuli into different intracellular cascades and networks.

## Materials and Methods

### Phytoplankton

The dinoflagellate *Alexandrium tamarense* clonal strain was isolated in May 2004 from the North Sea coast of Scotland [22], grown in K-medium [56] prepared from filtered North Sea water (0.2 µm; salinity ∼33‰) in a light-dark cycle (14 h:10 h) and diluted with K-medium to a concentration of ∼500 cells mL^−1^ prior to the experiment. The used clonal strain contains intermediate toxin content in comparison with 9 other *A. tamarense* strains (see “clone 5” in [57]).

### Zooplankton

Female copepods were collected with vertical WP2 net hauls (200 µm) from 50 m depth to the surface in the North Sea during 12–29 August 2007. *Oithona similis* and *Calanus helgolandicus* were collected off the coast of Scotland and *A. clausii* was collected off the northwest coast of Denmark. All copepods were starved for 24 hours in K-medium prepared from filtered seawater (0.2 µm; salinity ∼33‰) at 15°C in darkness before each experiment. To avoid anoxia in the 20 L containers the seawater was gently bubbled with air (5–10 cm between each bubble). Females were chosen because they release metabolites that attract males and function as a chemical trail potential perceived by *A. tamarense* in this experiment [58], [59].

### Grazing experiments

To investigate if the presence of copepods has an effect on the toxicity of *A. tamarense* we incubated the clonal strain in two types of K-medium prepared from filtered (0.2 µm) sea water collected *in situ*; **a**) K-medium with no further manipulation (control treatment) and **b**) K-medium in which copepods had been kept for 24 hours and potential released metabolites during the incubation (copepod-cue treatment). In each experiment *A. tamarense* was incubated in 1.15 L bottles at a concentration of approximately 4×10^6^ cells L^−1^. This concentration was necessary to ensure enough material for downstream analysis (PST determination and gene expression analysis). Nine bottles were incubated for each treatment, three bottles containing copepods, three bottles containing the K-medium with potential copepod-cues (treatments) and three bottles without copepods or any trait of them (control). For copepod containing bottles, we either used (per bottle) 10 *C. helgolandicus* females, 15 *A. clausii* females or 30 *O. similis* females. The K-medium was consequently pretreated with potential copepod cues from the corresponding number of copepod females. All copepod species used for this experiment were dominating species in the waters investigated. The feeding range of the selected copepod species is within the right range to enable them grazing on *A. tamarense* cells (3 µm up to several 100 µm for *C. helgolandicus* [60], [61], 18–60 µm for *A. clausii* [62] and 20–50 µm for *O.similis* [63]). We determined the ingestion rates of three different species of adult copepod females (*C. helgolandicus, A. clausii* and *O. similis*) feeding on *A. tamarense* in the treatments (see Figure 2). Incubations were run for 48 h in the dark on a plankton wheel rotating at ∼1 rpm at 15°C. Ingestion rates were calculated using the equations of Frost [64].

### Cell harvesting

Culture bottles were taken off the plankton wheel after 48 h, careful turned over head 7 times to ensure equal mixing of the culture. A sterile pipette tip was used to take out 10 mL of the culture and preserved in acid Lugol's solution for cell counts. Afterwards the culture was poured through a 100 µm mesh followed by a 10 µm mesh to filter out the copepods and to sample the cells. The copepods were transferred into a container with sterile filtered sea water for further examination of their condition. The 10 µm mesh containing the cells was washed out into a 50 mL collection tube with sterile sea water and adjusted to a volume of 40 mL. From this concentrated cell culture 4 mL were used for toxin analysis and the remaining 36 mL where immediately centrifuged at 4°C for 5 min for RNA samples. Cell pellets were immediately mixed with 1 mL 60°C hot TriReagent (Sigma-Aldrich, Steinheim, Germany), transferred to a 2 mL cyrovial containing acid washed glass beads. Cells were lysed using a Bio101 FastPrep instrument (Thermo Savant Illkirch, France) at maximum speed (6.5 m s^−1^) for 45 s. Afterwards, cells were frozen in liquid nitrogen and stored at −80°C until further use.

### Copepod condition

All copepods were individually examined under a stereomicroscope at the end of the experiment. The escape response of each copepod was assessed by observing its ability to avoid being drawn up into a pipette. If the copepods showed escape response and normal swimming behavior they were categorized as “not affected”. Copepods without any escape response and copepods without heart beat were categorized as “affected”. The proportion of affected versus non affected individuals was compared within the group for every species over all three replicates.

### Counting procedure

Lugol's fixed *Alexandrium* cells were counted after sedimentation of 3×1 mL aliquots using an inverted microscope. In both initial and final samples, all cells in the 1 mL were counted.

### RNA-Isolation

For RNA-Isolation, the suspended cell lysate was thawed on ice. After thawing, 200 µL of pure chloroform was added to each vial and vortexed for 15 s. The mixture was incubated for 10 min at room temperature and afterwards centrifuged for 15 min at 4°C with 10.000 g. The upper aqueous phase was transferred to a new vial, filled up with an equal volume of 100% isopropanol, vortexed and incubated for 2 hours at −20°C to precipitate the RNA. The RNA-pellet was collected by 20 min centrifugation at 4°C and 10.000 g. The pellet was washed with 70% EtOH, air dried and dissolved with 100 µL RNase free water (Qiagen, Hilden, Germany). The RNA-sample was further cleaned with the RNeasy Kit (Qiagen) according to manufactures protocol for RNA clean-up including on-column DNA-digestion. RNA quality check was performed using a NanoDrop ND-100 spectrometer (PeqLab, Erlangen, Germany) for purity and the RNA Nano Chip Assay with the 2100 Bioanalyzer device (Agilent Technologies, Böblingen, Germany) was just to examine the integrity of the extracted RNA. Just high quality RNAs (OD 260/280>2 and OD260/230>1.8) as well as with intact ribosomal peaks (obtained from the Bioanalyzer readings) were used for further experiments.

### Gene expression analysis using microarrays

*A. tamarense* total RNA samples derived from the copepod treatment and the waterborne cues treatment were hybridized against the respective control treatment. The microarray hybridization procedure was carried out using 500 ng total RNA from each sample and the Agilent two color low RNA Input Linear Amplification kit (Agilent Technologies, Waldbronn, Germany). Prior to the labeling, the Agilent RNA Spike-In Mix (Agilent) was added to the RNA. The RNA was reverse transcribed into cDNA, amplified towards cRNA and labeled following the ‘Agilent Low RNA Input Linear Amplification Kit’ protocol (Agilent). Dye incorporation rates (cyanine-3 and cyanine-5) and cRNA concentrations were measured using the NanoDrop ND-100 spectrometer (PeqLab). Hybridization was performed onto 4x44k microarray slides at 65°C for 17 h (Agilent). The microarrays contained 60mer oligonucleotide probes designed by Agilent's E-array online platform from three *Alexandrium* spp. EST libraries. For each hybrization, 825 ng of cyanine-3 and cyanine-5 labeled cRNA were used. After hybridization, microarrays were disassembled and washed according to manufacturer's instructions (Agilent). Microarrays were scanned using an Agilent G2565AA scanner. Raw data were processed with the Agilent Feature Extraction Software version 9.1.3.1 (FE). Array quality was monitored using the Agilent QC Tool (v1.0) with the metric set GE2_QCMT_Feb07. The array design, raw data and normalized data and the detailed experimental design are MIAME compliant and deposited in a MIAME compliant database (ArrayExpress at the EBI; http://www.ebi.ac.uk/microarray-as/ae/; ID: E-MEXP-2874). Testing for differential expressed genes was performed using the GeneSpring GX software platform version 11 (Agilent) with the implemented T-test against zero statistics combining biological replicates. Genes were considered to be differential expressed when test statistics reply p-Values were less than 0.05 and calculated fold changes between the control and the treatment exceeded 1.5.

### PST-toxin analysis

PSP toxins were extracted according to Krock et al. [65]. The 4 mL concentrated cell solution was centrifuged for 15 min at maximum speed. The water was poured out and the cell pellet was transferred with 1 mL of sterile seawater to a 2 mL tube and centrifuged for another 10 min at maximum speed. The seawater was subsequently removed with a pipette and the pellet was transferred with 2×250 µL of acetic acid (0.03 N) into a FastPrep tube (Thermo Savant, Illkirch, France) containing 0.9 g of lysing matrix D. Cells where lysed by reciprocal shaking in a Bio101 FastPrep instrument (Thermo Savant) at maximum speed (6.5 m s^−1^) for 45 s. and centrifuged afterwards for 15 min at 13.000 g and 4°C. From the supernatant, 400 mL were passed through a spin filter (pore size 0.45 µm) by centrifugation for 30 sec. at 3000 g. For the copepods the same procedure was applied with the following chances: after removing the remaining seawater from the copepods, some liquid nitrogen was filled in the tube and the copepods were crushed. After evaporation of the nitrogen the acetic acid was filled in the tube. The samples were further processed as described for the cell pellet samples. The filtrate was injected into the HPLC/FLD equipped with a fluorescence detector The LC-FD analysis was carried out as previously described in detail in Krock et al [65] and Yang et al [66].

## Supporting Information

Table S1 **Full list of regulated ESTs with annonations from the *C. heloglandicus* treatments with live copepods and respective cues.**
(XLS)

Table S2 **Full list of regulated ESTs with annonations from the *O. similis* treatments with live copepods and respective cues.**
(XLS)

Table S3 **Full list of regulated ESTs with annonations from the *A.clausii* treatments with live copepods and respective cues.**
(XLS)
